# An optimized deep learning model based on transperineal ultrasound images for precision diagnosis of female stress urinary incontinence

**DOI:** 10.3389/fmed.2025.1564446

**Published:** 2025-04-28

**Authors:** Ke Chen, Qi Chen, Ning Nan, Lu Sun, Miaoyan Ma, Shanshan Yu

**Affiliations:** ^1^Department of Ultrasound, The Second Affiliated Hospital of Xi'an Jiaotong University, Xi'an, China; ^2^Department of Urology, The Second Affiliated Hospital of Xi'an Jiaotong University, Xi'an, China

**Keywords:** stress urinary incontinence, deep learning, transperineal ultrasound, diagnostic accuracy, Densenet-121

## Abstract

**Background:**

Transperineal ultrasound (TPUS) is widely utilized for the evaluation of female stress urinary incontinence (SUI). However, the diagnostic accuracy of parameters related to urethral mobility and morphology remains limited and requires further optimization.

**Objective:**

This study aims to develop and validate an optimized deep learning (DL) model based on TPUS images to improve the precision and reliability of female SUI diagnosis.

**Methods:**

This retrospective study analyzed TPUS images from 464 women, including 200 patients with SUI and 264 controls, collected between 2020 and 2024. Three DL models (ResNet-50, ResNet-152, and DenseNet-121) were trained on resting-state and Valsalva-state images using an 8:2 training-to-testing split. Model performance was assessed using diagnostic metrics, including area under the curve (AUC), accuracy, sensitivity, and specificity. A TPUS-index model, constructed using measurement parameters assessing urethral mobility, was used for comparison. Finally, the best-performing DL model was selected to evaluate its diagnostic advantages over traditional methods.

**Results:**

Among the three developed DL models, DenseNet-121 demonstrated the highest diagnostic performance, achieving an AUC of 0.869, an accuracy of 0.87, a sensitivity of 0.872, a specificity of 0.761, a negative predictive value (NPV) of 0.788, and a positive predictive value (PPV) of 0.853. When compared to the TPUS-index model, the DenseNet-121 model exhibited significantly superior diagnostic performance in both the training set (*z* = −2.088, *p* = 0.018) and the testing set (*z* = −1.997, *p* = 0.046).

**Conclusion:**

This study demonstrates the potential of DL models, particularly DenseNet-121, to enhance the diagnosis of female SUI using TPUS images, providing a reliable and consistent diagnostic tool for clinical practice.

## Introduction

Stress urinary incontinence (SUI) refers to the involuntary leakage of urine during activities that increase intra-abdominal pressure, such as coughing or physical exertion. It is commonly observed in female, significantly impacting the quality of their life. Studies have reported that the prevalence of SUI in postmenopausal women ranges from 10% to 40% (1). From the pathophysiological perspective, the development of SUI is primarily associated with damage to the supportive structures of the bladder neck and proximal urethra, as well as excessive urethral mobility (2).

Transperineal ultrasound (TPUS) is widely utilized in clinical practice to evaluate pelvic floor dysfunction, including SUI. This non-invasive imaging modality provides clear visualization of pelvic floor structures, such as the urethra, bladder, and vagina, and enables quantitative assessment of urethral mobility (3, 4). Measurable parameters derived from TPUS, including bladder neck descent (BND), urethral rotation angles (URA), and urethral length, hold diagnostic value for female SUI (5–7). However, current ultrasound techniques for diagnosing SUI face significant challenges. The dependence of TPUS on operator experience may lead to inconsistencies in diagnostic results, affecting clinical decision-making. Patient cooperation directly impacts the accuracy of parameters, such as the intensity and duration of the Valsalva maneuver. Moreover, the dynamic changes in the structures surrounding the urethra are complex, and existing parameters may overlook certain important functional abnormalities, resulting in incomplete diagnosis. These limitations underscore the need for innovative approaches to enhance the accuracy and reliability of SUI diagnosis.

Recent advancements in artificial intelligence (AI), particularly deep learning (DL) algorithms, have demonstrated significant potential in enhancing diagnostic accuracy in medical imaging. Unlike traditional machine learning methods, DL models automatically extract detailed structural features from raw data without requiring operator expertise or manually designed feature extraction (8). DL algorithms exhibit exceptional proficiency in segmenting pelvic floor ultrasound images and identifying pelvic floor structures. Additionally, they are capable of dynamically segmenting and automatically measuring anterior pelvic structures, such as bladder neck descent and urethral rotation angles (9–12). These capabilities make DL models potentially capable of achieving breakthroughs in addressing the limitations of conventional TPUS diagnostics for female SUI.

This study aims to develop and validate a convolutional neural network (CNN)-based DL model optimized for TPUS imaging to overcome key challenges in diagnosing SUI. By harnessing DL's capabilities for automated feature extraction and precise analysis, the model strives to enhance diagnostic accuracy, while reducing reliance on operator expertise and mitigating measurement variability. Ultimately, this study seeks to establish a diagnostic tool that can be integrated into clinical workflows, facilitating earlier detection and improved management of SUI.

## Materials and methods

### Objects

The study retrospectively collected data from female patients who underwent TPUS examinations at the Department of Urology and Gynecology, the Second Affiliated Hospital of Xi'an Jiaotong University from 2020 to 2024. The study was conducted in compliance with the Declaration of Helsinki and approved by the Institutional Review Board (IRB number 2020823).

**Figure 1:**
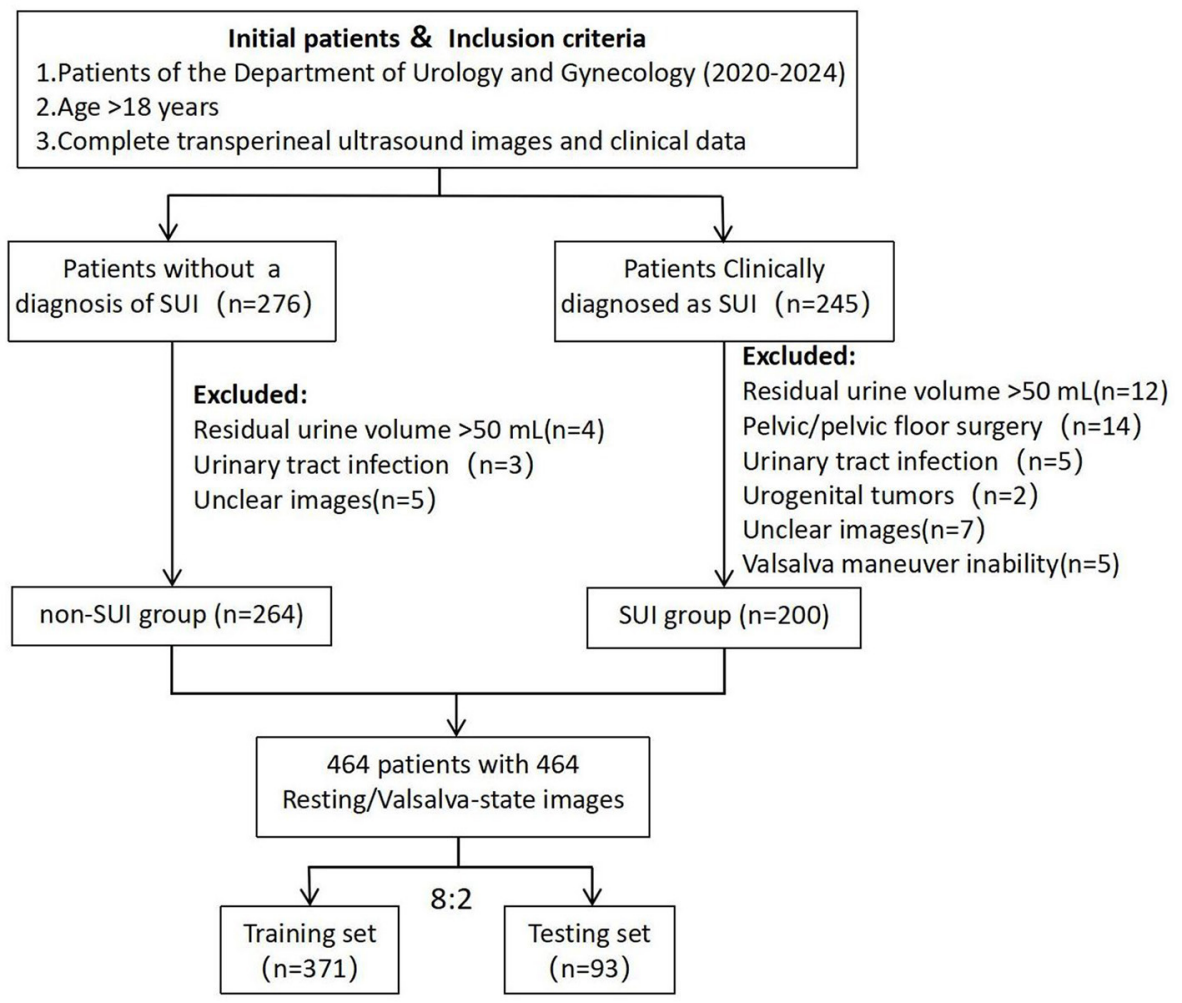
The flow diagram of recruitment and grouping of research objects.

All patients underwent a comprehensive clinical evaluation, which included completing the International Consultation on Incontinence Questionnaire-Urinary Incontinence Short Form (ICIQ-UI-SF), the International Consultation on Incontinence Questionnaire-Female Lower Urinary Tract Symptoms (ICIQ-FLUTS), urinalysis, uroflowmetry, and maintaining a 3-day bladder diary (13, 14). Inclusion criteria for the SUI group were: (1) age >18 years; (2) clinical diagnosis of SUI; (3) availability of complete TPUS images and clinical data. Exclusion criteria included: (1) residual urine volume >50 mL; (2) history of pelvic or pelvic floor reconstructive surgery; (3) active urinary tract infection or history of urogenital tumors; (4) unclear ultrasound images or inability to perform the Valsalva maneuver. A total of 200 patients meeting these criteria were included in the SUI group. Additionally, 264 female patients without a diagnosis of SUI during the same period, who fulfilled the inclusion criteria, were selected as the control group (non-SUI group). Transperineal ultrasound images were collected for all patients, including 464 resting-state images and 464 Valsalva-state images (Figure 1).

### Ultrasound images acquisition

**Figure 2:**
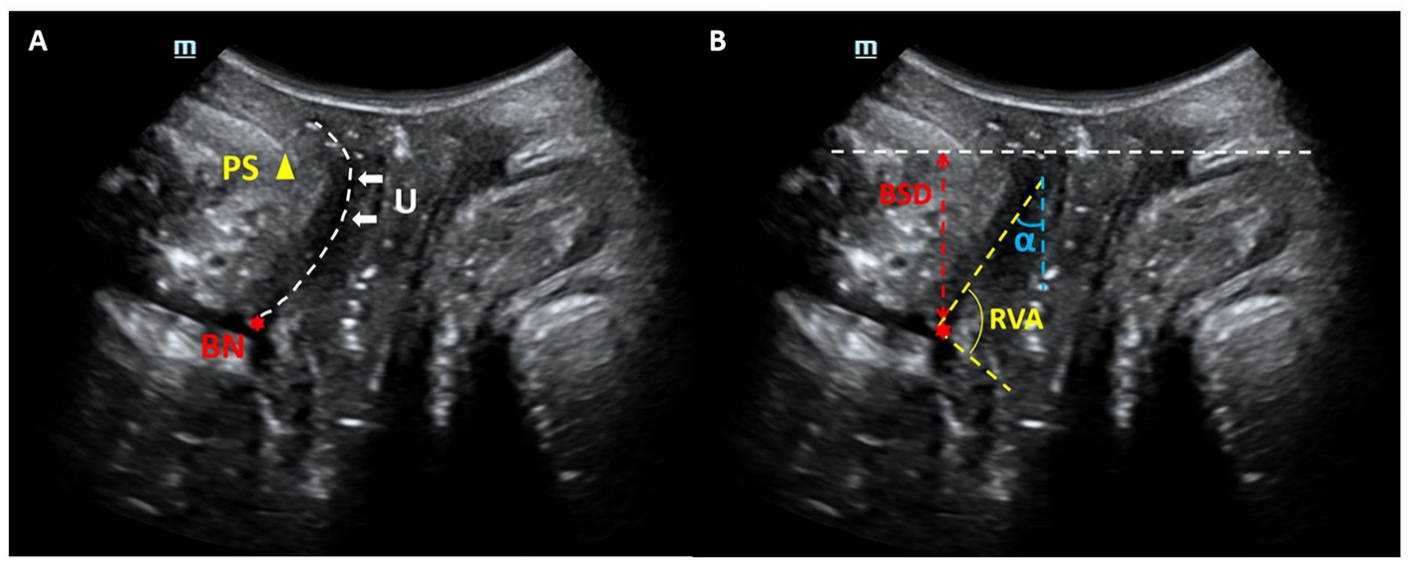
Mid-sagittal ultrasound images of the pelvic organs. **(A)** Anatomical landmarks: the public symphysis (PS), bladder neck (BN), and urethra (U) are also visualized. **(B)** Index measurement mark: bladder symphyseal distance (BSD), urethral axis angle (α angle) and retrovesical angle (RVA).

TPUS examinations were conducted by expert sonographer with 5–10 years of the pelvic for ultrasound experience using a Mindray Resona 8 ultrasound system equipped with a DE10-3U 3D volumetric probe (frequency range: 3–10 MHz). Patients were positioned in the lithotomy position after bladder emptying, following the protocol established by Dietz HP (15). Mid-sagittal pelvic floor static images were obtained at rest and during the Valsalva maneuver. Key parameters, including bladder symphyseal distance (BSD), urethral axis angle (α angle), and retrovesical angle (RVA), were measured. Additionally, bladder neck descent (BND) and urethral rotation angle (URA) were calculated. Ultrasound static images were exported in JPG format for subsequent analysis (Figure 2).

### Deep learning model development

Ultrasound images in JPG format were imported into the MedAI Darwin learning platform (http://premium.darwin.yizhun-ai.com). Using the platform's tools, the urethra was delineated as the region of interest (ROI). The annotation process was carried out by an experienced pelvic floor ultrasound specialist, and any disagreements were resolved through consensus discussion. The dataset included 928 images (464 resting-state and 464 Valsalva-state), with annotated ROIs. The annotated data were randomly divided into a training set (*n* = 371) and a testing set (*n* = 93) in an 8:2 ratio. Following this, preprocessing operations such as data augmentation and normalization were performed on the input ROI sub-images, including random flipping, image transposition, and pixel value normalization (16).

**Figure 3:**
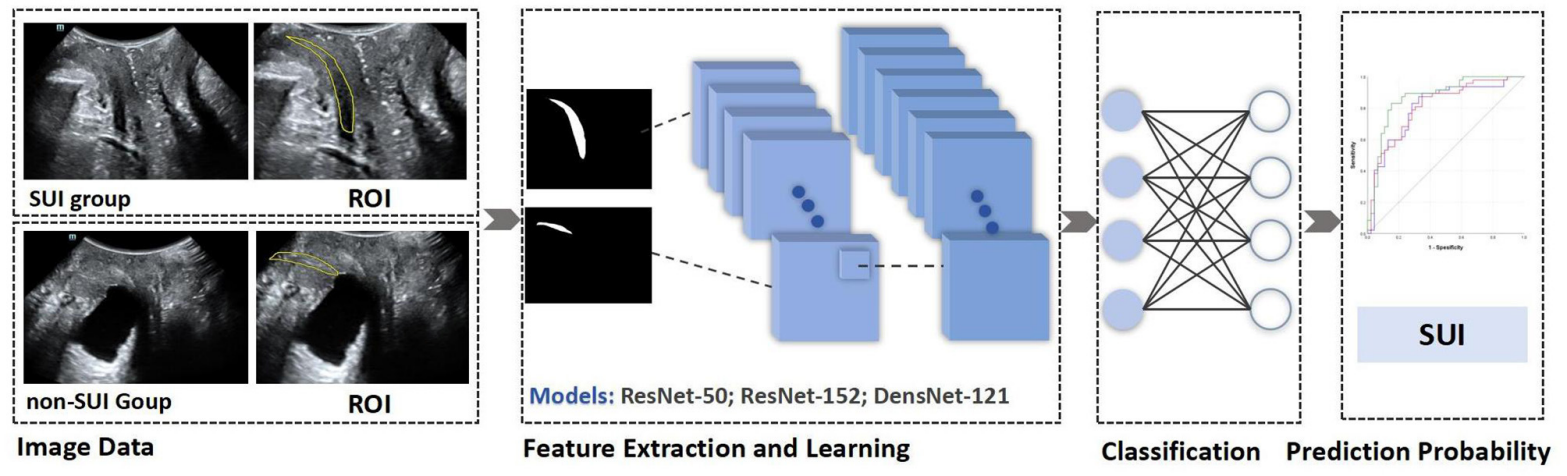
Schematic diagram of DL models in predicting the risk of SUI. DL, Deep Learning; TPUS, Transperineal Ultrasound; SUI, Stress Urinary Incontinence.

Three DL architectures were implemented for model development: ResNet-50, ResNet-152, and DenseNet-121. The models were trained separately on resting-state and Valsalva-state images to predict the presence of SUI. Model performance was evaluated using standard diagnostic metrics, including the area under the receiver operating characteristic curve (AUC), accuracy, sensitivity, specificity, positive predictive value (PPV), and negative predictive value (NPV). Receiver operating characteristic (ROC) curves were generated to visualize and compare the classification performance of the models. The complete experimental workflow is shown in Figure 3.

### Construction of the TPUS-index model in predicting the risk of SUI

A TPUS-index model was constructed using ultrasonic measurement parameters for assessing urethral morphology and mobility as independent variables, with SUI diagnosis as the outcome variable. Binary logistic regression analyses were performed to identify significant predictors of SUI, and the model's predictive performance was evaluated using AUC, accuracy, sensitivity, and specificity.

### Statistical analysis

Statistical analyses were conducted using SPSS software (version 26.0; IBM Corp). Continuous variables were expressed as mean ± standard deviation (SD) and compared using independent samples *t*-tests. Categorical variables were presented as counts and percentages and analyzed using chi-square tests. Univariate and multivariate logistic regression with forward stepwise analysis were applied to screen for independent risk factors and establish a TPUS-index model. The predictive performance of the model was evaluated by plotting ROC curves and calculating the AUC along with the consistency index (CI). Comparisons of AUC values between models were performed using *z*-tests. All statistical tests were two-tailed, and a *p-value* < 0.05 was considered statistically significant.

## Results

### Baseline characteristics

The baseline characteristics of the study population are summarized (Supplementary Table 1). The mean age of the participants was 47.90 ± 14.88 years, ranging from 19 to 90 years. The average parity was 1.38 ± 0.80, and the mean BMI was 23.22 ± 1.80 kg/m^2^. Among the participants, 233 women (50.2%) were postmenopausal. The clinical characteristics were balanced between the testing set and the training set, with no significant differences in age, parity, BMI, or menopausal status.

### Diagnostic performance of DL models

The DL models based on resting-state images exhibited inferior diagnostic performance. In contrast, the DL models trained on Valsalva-state images demonstrated significantly better performance in predicting SUI. DenseNet-121 achieved the best discriminatory ability among the three models, with a well-balanced performance across multiple diagnostic metrics, including accuracy (81.7%), sensitivity (87.2%), and specificity (76.1%) (Table 1).

**Table 1:** Comparison of the diagnostic performance between DL models for SUI based on TPUS images.

**Image mode**	**DL model**	**Group**	**AUC(95%CI)**	**Accuracy**	**Sensitivity**	**Specificity**	**PPV**	**NPV**
Resting	ResNet-50	Training set	0547 (0.487, 0.606)	0.578	0.726	0.380	0.611	0.508
		Testing set	0.605 (0.490, 0.720)	0.559	0.269	0.927	0.824	0.500
	ResNet-152	Training set	0.482 (0.421, 0.542)	0.589	0.948	0.108	0.588	0.607
		Testing set	0.553 (0.436, 0.670)	0.548	0.250	0.927	0.813	0.494
	DensNet-121	Training set	0.532 (0.472, 0.591)	0.538	0.392	0.734	0.664	0.473
		Testing set	0.490 (0.369,0.610)	0.516	0.192	0.927	0.769	0.475
Valsalva	ResNet-50	Training set	0.713 (0.659, 0.766)	0.695	0.757	0.608	0.733	0.637
		Testing set	0.803 (0.710, 0.896)	0.774	0.830	0.717	0.750	0.805
	ResNet-152	Training set	0.761 (0.712, 0.810)	0.722	0.739	0.699	0.778	0.652
		Testing set	0.809 (0.720, 0.898)	0.763	0.872	0.652	0.719	0.833
	DensNet-121	Training set	0.798 (0.752, 0.845)	0.747	0.761	0.725	0.832	0.681
		Testing set	0.869 (0.793, 0.945)	0.817	0.872	0.761	0.788	0.853

DL, Deep Learning; AUC, Area Under the ROC Curve; CI, Confidence Interval; PPV, Positive Predictive Value; NPV, Negative Predictive Value.

**Figure 4:**
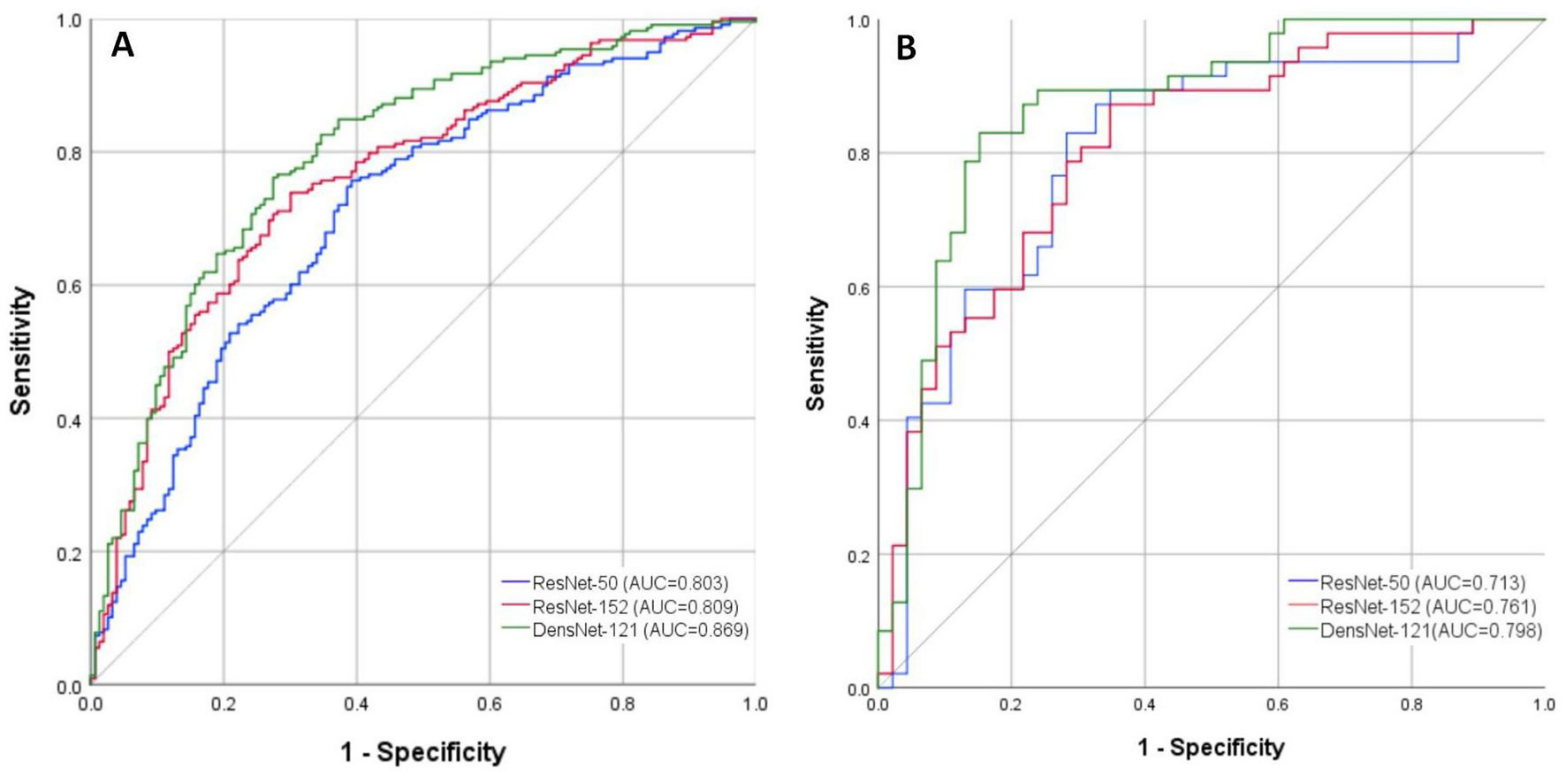
ROC curves of the DL models for diagnosing SUI based on Valsalva-state TPUS images. **(A)** Training Set; **(B)** Testing Set. ROC, Receiver Operating Characteristic; DL, Deep Learning; SUI, Stress Urinary Incontinence; TPUS, Transperineal Ultrasound.

In the training set, significant differences were found between ResNet-50 and ResNet-152 (*z* = −2.149, *p* = 0.032) and between ResNet-50 and DenseNet-121 (*z* = −3.568, *p* < 0.001). However, no significant difference in AUC values was observed between ResNet-152 and DenseNet-121 (*z* = −1.661, *p* = 0.097). In the testing set, DenseNet-121 demonstrated a statistically significant superiority in AUC compared to ResNet-152 (*z* = −2.372, *p* = 0.029) and ResNet-50 (z = −2.190, *p* = 0.018). These results confirm the superior classification ability of DenseNet-121 in both training and testing datasets (Figure 4).

### Construction of the TPUS-index model in predicting the risk of SUI

Among the TPUS measurement parameters, BSD-rest and BSD-valsalva were lower in the SUI group (OR: 0.910 and 0.894, *p* < 0.001), while α angle-valsalva, BND, and URA were higher in the SUI group (OR: 1.037, 1.124, and 1.023, *p* < 0.001). Additionally, α angle-rest was slightly lower in the SUI group (OR: 0.988, *p* = 0.015; Table 2). Based on the univariate analysis, six significant variables (BSD-rest, BSD-valsalva, α angle-rest, α angle-valsalva, BND, and URA) were included in the multivariate logistic regression analysis due to their strong statistical association with SUI (*p* < 0.05).

**Table 2:** Univariate binary logistic regression analysis of TPUS ultrasonic measurement.

**TPUS measurement variable**	**SUI group (*n* = 200)**	**non SUI group (*n* = 264)**	**Odds ratio (95% CI)**	***P-value***
BSD-rest(mm, mean ± SD)	22.460 ± 4.783	23.980 ± 3.465	0.910 (0.867, 0.956)	0.000
BSD-valsalva(mm, mean ± SD)	−1.310 ± 11.462	14.440 ± 7.862	0.894 (0.873, 0.916)	0.000
α angle-rest(°, mean ± SD)	24.690 ± 17.335	28.980 ± 19.646	0.988 (0.978, 0.998)	0.015
α angle-valsalva(°, mean ± SD)	45.600 ± 25.337	25.050 ± 20.535	1.037 (1.028, 1.046)	0.000
RVA-valsalva(°, mean ± SD)	137.180 ± 24.936	133.440 ± 20.467	1.008 (0.999, 1.016)	0.079
BND(mm, mean ± SD)	23.770 ± 10.552	14.440 ± 7.862	1.124 (1.095, 1.153)	0.000
URA(°, mean ± SD)	59.340 ± 29.929	40.900 ± 26.633	1.023 (1.016, 1.030)	0.000

TPUS, Transperineal Ultrasound; SUI, Stress Urinary Incontinence; CI, Confidence Interval; BSD, Bladder Symphyseal Distance; RVA, Retrovesical Angle; BND, Bladder Neck Descent; URA, Urethral Rotation Angle; SD, Standard Deviation.

The Rad-score of the TPUS-index model achieved an AUC of 0.736 (95% CI: 0.629 – 0.843), with an accuracy, sensitivity, and specificity of 77.2%, 67.5%, and 75.5%, respectively (Table 3). The model formula is as follows: RadScore = −0.047^*^BSD(valsalva) – 0.036^*^BSD(rest) + 0.020^*^α angle(valsalva) + 0.018^*^BND −0.008^*^α angle(rest) −0.002^*^URA −0.003.

**Table 3:** In comparison of the performance between DL model and TPUS-index model in predicting SUI in the training and testing sets.

**Group**	**Diagnostic model**	**AUC (95% CI)**	***P-value***	**Accuracy**	**Sensitivity**	**Specificity**	**PPV**	**NPV**
Training set	DL Model	0.798 (0.752, 0.845)	0.018	0.747	0.761	0.725	0.832	0.681
	TPUS-index Model	0.721 (0.666, 0.777)		0.730	0.631	0.806	0.711	0.742
Testing set	DL Model	0.869 (0.793, 0.945)	0.046	0.817	0.872	0.761	0.788	0.853
	TPUS-index Model	0.736 (0.629, 0.843)		0.772	0.675	0.755	0.675	0.775

DL, Deep Learning; TPUS, Transperineal Ultrasound; SUI, Stress Urinary Incontinence; AUC, area under the ROC curve; PPV, Positive Predictive Value; NPV, Negative Predictive Value.

### Comparison of the diagnostic performance between DL model and TPUS-index model

In the training set, the DL model demonstrated a significantly higher AUC compared to the TPUS-index model (*z* = −2.088, *p* < 0.05), indicating superior diagnostic performance. Similarly, in the testing set, the AUC of the DL model was also significantly higher than that of the TPUS-index model (*z* = −1.997, *p* < 0.05). Beyond the AUC, other diagnostic metrics, including accuracy, sensitivity, and specificity, was consistently better for the DL model compared to the TPUS-index model. This highlights the DL model's enhanced capability in diagnosing SUI, offering a more robust and reliable diagnostic approach compared to the TPUS-index model (Table 3 and Supplementary Figures 1, 2).

## Discussion

This study developed a DL model for the diagnosis of female SUI and compared its diagnostic performance with that of an ultrasound assessment model. Our findings demonstrate that the DL model outperformed the TPUS-index model in predicting the disease.

Our findings revealed that DL models trained on resting-state images exhibited poor diagnostic performance. In contrast, models trained on Valsalva-state images demonstrated significantly better performance in diagnosing SUI. The inferior performance of resting-state models can be attributed to the lower recognition rate of organs farther from the probe, such as the bladder and uterus, by CNN under resting conditions (9). Additionally, the morphology and function of the urethra change during the Valsalva maneuver. The limited anatomical changes observable under resting conditions provide insufficient diagnostic information for the model to learn effectively (17). This aligns with current clinical practice, where sonographer prioritize changes in the urethral angle and position in Valsalva-state images when assessing SUI through pelvic floor ultrasound (2). These findings indicate that DL models relying solely on resting TPUS images lack the reliability required for accurate diagnosis of female SUI.

Among the three DL models developed using Valsalva-state images, DenseNet-121 outperformed the ResNet models (ResNet-152 and ResNet-50), particularly in the testing set. This superior performance highlights DenseNet-121′s network architecture, which offers enhanced feature reuse and information flow through its dense connections, significantly reducing parameter redundancy. This optimization makes DenseNet-121 more efficient in terms of both parameter count and computational performance. The model exhibits strong classification capabilities in disease diagnosis through medical imaging (18–20). In the testing set, DenseNet-121 successfully identified four positive cases of SUI that were missed by ResNet-50 and ResNet-152. This superior sensitivity underscores DenseNet-121′s ability to capture subtle features in images. In contrast, the limitations of the ResNet models stem from their residual connections being confined to adjacent layers, which results in less effective feature reuse and greater computational resource requirements during training.

TPUS is a non-invasive and repeatable tool commonly used to assist in the assessment of female SUI. The SUI prediction models established based on TPUS ultrasound measurement data are also one of the current research hotspots. Liu and Quan (21) developed a postpartum SUI model using clinical data, bladder neck descent, and urethral funneling, achieving an AUC of 0.807 in the validation cohort. Another study on predicting SUI based on pelvic floor ultrasound data reported that combining multiple measurement parameters in the model achieved an AUC of 0.802, with sensitivity ranging from 0.542 to 0.665 and specificity from 0.867 to 0.980 (6). This study also demonstrated the predictive value of ultrasound parameters in diagnosing SUI. Indicators of Valsalva-State had a significant impact on the model's Rad-Score, consistent with previous research findings. These results further emphasize the critical role of Valsalva maneuver images in evaluating SUI. However, the limitations of such models lie in the need to collect clinical information or repeatedly measure multiple ultrasound parameters, which is time-consuming and highly dependent on the operator's technical expertise and dynamic observation skills.

This study found that the optimal DL model developed using TPUS images demonstrated higher diagnostic value for SUI compared to the TPUS-index model. The advantages of DL models lie in their ability to capture subtle imaging details that are challenging for sonographers while performing rapid automated analysis, reducing human errors and significantly shortening operational time. Notably, the optimal DL model in our study showed a significant advantage in sensitivity compared to the TPUS-index model. However, five positive cases in the testing set were misclassified by the DL model. Upon analysis, three cases involved uterine prolapse. Since uterine prolapse can cause structural changes in the adjacent urethra and posterior bladder wall, it may have affected the model's automatic segmentation of the urethra, thereby influencing the classification results. In addition, the DenseNet-121 model shows suboptimal performance with a specificity of 0.761 in ruling out non-SUI cases. Possible reasons include the limited number of non-SUI cases in the dataset, which may cause the model to be biased toward the majority class, resulting in insufficient ability to identify non-SUI cases. Additionally, non-SUI cases may exhibit diverse manifestations, making it difficult for the model to capture their complex features. To address these issues, improvements can be made by balancing the dataset and enhancing data diversity, or by fine-tuning the model or customizing layers to better capture the features of non-SUI cases. Despite these challenges, the DL model exhibited excellent performance in AUC and sensitivity for SUI diagnosis, making it a valuable tool for early detection. With an aging population, the rising incidence of SUI and its impact on quality of life have gained widespread attention. Early diagnosis and intervention are crucial in managing SUI. Accurate identification of early-stage SUI patients and the provision of timely treatments, such as lifestyle modifications, pelvic floor muscle therapy, and pharmacological interventions, can effectively slow disease progression. Compared to late-stage surgical treatments, these measures are more cost-effective, less invasive, and significantly improve patients' quality of life. Furthermore, the development of DL models facilitates large-scale screening for this condition in the general population.

## Limitations of the study

First, the sample size included in this study was relatively small, necessitating further research with larger, more diverse prospective samples. Importantly, external validation in diverse populations, such as multi-center cohorts or across varying ultrasound devices, is currently absent and represents a critical limitation. Future steps should include multi-center validation and testing across different imaging devices to ensure the model's adaptability and reliability in various clinical settings. Second, the developed DL model analyzed only ultrasound images and did not integrate clinical data. Therefore, in the next phase, we plan to combine clinical data with the DL model to design and construct a new hybrid model, further improving its diagnostic performance for female SUI.

## Conclusion

In summary, this study developed a DL model for diagnosing female SUI, showing significant improvements in specificity, sensitivity, accuracy, and testing set consistency compared to the TPUS-index models, and its diagnostic performance was validated. It demonstrates the potential of DL models to enhance diagnostic accuracy and automation for female SUI.

## Data Availability

The original contributions presented in the study are included in the article/Supplementary material, further inquiries can be directed to the corresponding author.
