# Supplementary material for: An optimized deep learning model based on transperineal ultrasound images for precision diagnosis of female stress urinary incontinence
Source: Front Med (Lausanne). 2025 Apr 28;12:1564446. doi: 10.3389/fmed.2025.1564446 (PMC12066636; doi:10.3389/fmed.2025.1564446)
Supplement: Supplementary file 1 [file Data_Sheet_1.zip › Supplementary files/Supplementary Table.docx]

**Supplementary Table 1** Comparison of clinical characteristics between the testing set and the training set

| Characteristics | Total (n=464) | Training set (n=371) | Testing set (n=93) | P*-*value |
| --- | --- | --- | --- | --- |
| Age (years, mean±SD) | 47.90±14.88 | 47.97±15.10 | 47.63±14.05 | 0.166 |
| Parity( mean±SD) | 1.38±0.80 | 1.39±0.81 | 1.38±0.77 | 0.739 |
| BMI(kg/m^2^, mean±SD) | 23.22±1.80 | 23.17±1.76 | 23.43±1.92 | 0.570 |
| Menopause, N(%) | 233（50.22） | 183（49.32） | 45（48.39） | 0.693 |

SD, standard deviation; BMI, Body Mass Index.
