# Supplementary figures and images for: An optimized deep learning model based on transperineal ultrasound images for precision diagnosis of female stress urinary incontinence
Source: Front Med (Lausanne). 2025 Apr 28;12:1564446. doi: 10.3389/fmed.2025.1564446 (PMC12066636; doi:10.3389/fmed.2025.1564446)

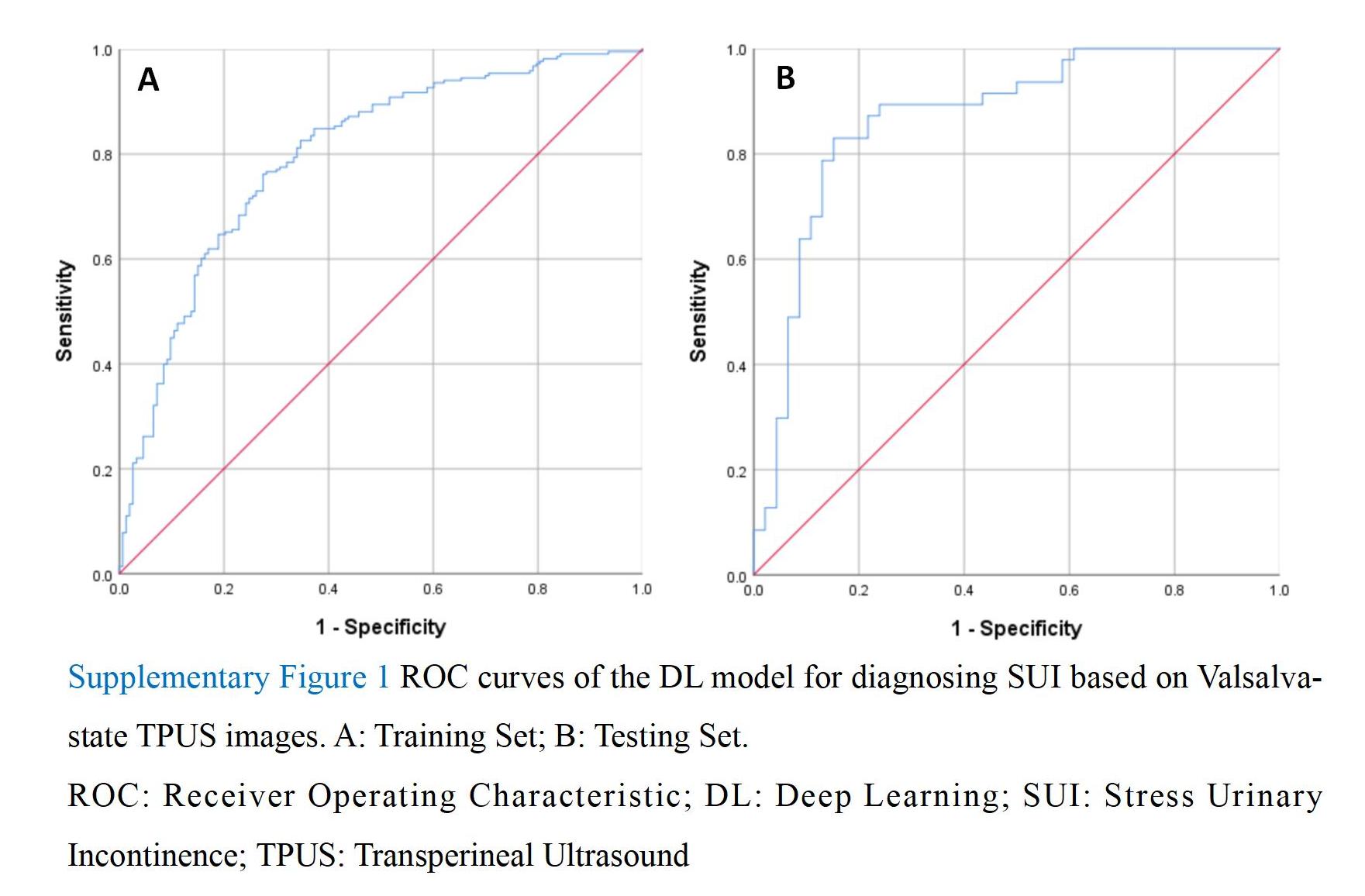

Supplement: Supplementary file 1 [file Data_Sheet_1.zip › Supplementary files/Supplementary Figure 1.jpg]

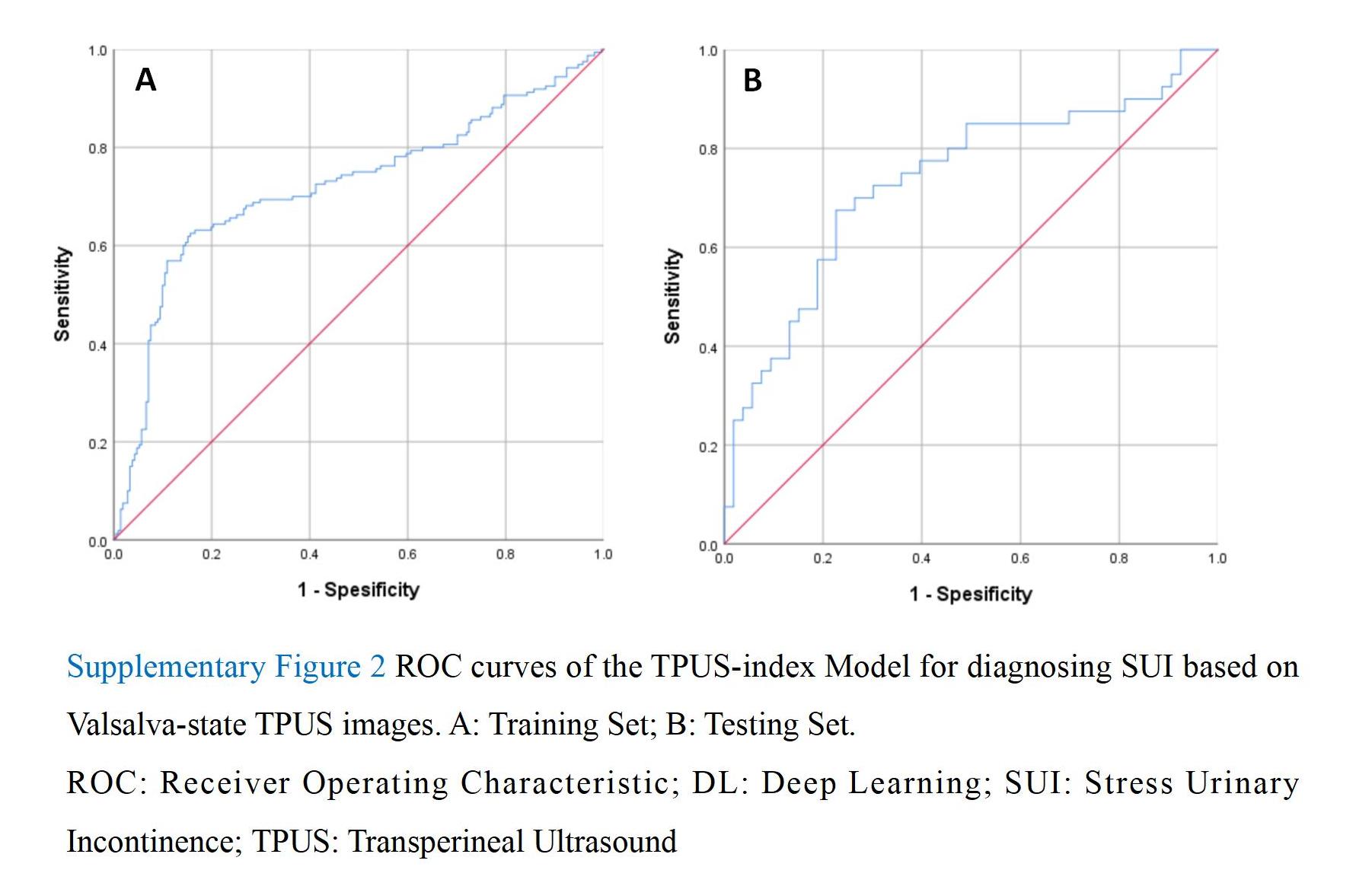

Supplement: Supplementary file 1 [file Data_Sheet_1.zip › Supplementary files/Supplementary Figure 2.jpg]
